# COVID‐associated arthritis after severe and non‐severe COVID‐19: A systematic review

**DOI:** 10.1002/iid3.1035

**Published:** 2023-10-11

**Authors:** Mahsa Zarpoosh, Parsa Amirian

**Affiliations:** ^1^ Kermanshah University of Medical Science (KUMS) Kermanshah Iran

**Keywords:** COVID‐19 severity, COVID‐associated arthritis, inflammatory arthritis, reactive arthritis

## Abstract

**Aim:**

Since the coronavirus outbreak became a global health emergency in 2020, various immune‐based effects, such as inflammatory arthritis (IA), have been recorded. This study aimed to determine the role of COVID‐19 severity on post‐COVID arthritis.

**Methods:**

We systematically reviewed 95 patients who developed arthritis after severe and non‐severe COVID‐19 infection by searching the databases, including PubMed, SCOPUS, and EMBASE. We used the term “COVID‐associated arthritis” because there was no definite diagnostic method for classifying arthritides after COVID‐19 infection, and the diagnosed arthritis types were based on the authors' viewpoints.

**Results:**

After evaluating the data between the two severe and non‐severe COVID‐19‐infected groups of patients, the results showed that the COVID‐19 severity may affect the pattern of joint involvement in IA. In both groups, combination therapy, including oral nonsteroidal anti‐inflammatory drugs with different types of corticosteroids, was the most common treatment. In addition, the mean age and comorbidities rate was higher in the severe COVID‐19 group. Even though the patients in the severe COVID‐19 group developed more serious COVID‐19 symptoms, they experienced milder arthritis with better outcomes and more delayed onsets that required less aggressive therapy.

**Conclusion:**

We conclude that there may be an inverse relationship between COVID‐19 severity and arthritis severity, possibly due to weaker immunity conditions following immunosuppressant treatments in patients with severe COVID‐19.

## Graphical abstract

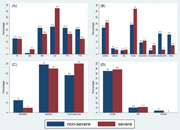

## 1 INTRODUCTION

Since the reconvention of The Emergency Committee on the novel coronavirus on January 30, 2020, and the declaration of the (2019‐nCoV) Outbreak to be a public health emergency, extreme measures have been taken place to understand the effects of SARS‐CoV‐2 virus (severe acute respiratory syndrome coronavirus 2) on the body and especially on the immune system.^1^ Rheumatic manifestations and immune‐mediated complications of the coronavirus disease have been studied extensively; these studies have found that SARS‐CoV‐2 may trigger the cascade of inflammatory mediators or be the primary agent for musculoskeletal manifestations, particularly inflammatory arthritis (IA).^2^, ^3^, ^4^

IA is non‐septic arthritis that includes conditions in which the body's defensive mechanisms attack joint tissues rather than germs or viruses. Common types of IA comprise reactive arthritis (Re‐A), rheumatoid arthritis (RA), ankylosing spondylitis (AS), psoriatic arthritis (PA), and gout arthritis (GA).^5^ Nearly 1% of all cases of acute IA are considered to have a viral etiology.^6^ Re‐A is the most common type of COVID‐associated IA that occurs as a “reaction” to an infection elsewhere in the body. Re‐A often appears in patients without a history of rheumatic and musculoskeletal diseases (RMDs). It may only be presented with peri‐articular manifestations such as tenosynovitis, tendinitis, enthesitis, dactylitis, and bursitis or in conjunction with arthritis.^7^, ^8^

Viral infections such as the SARS‐CoV‐2 virus can solely trigger musculoskeletal manifestations with a nonimmune pathway by directly invading joint tissues and cells; this event is called viral arthritis. Diagnosing and confirming viral arthritis can be challenging because, up to this date, there are no accepted diagnostic criteria to distinguish viral arthritis from post‐viral Re‐A.^8^ Our study used the term “COVID‐associated arthritis” interchangeably to include both viral arthritis and post‐viral IA.

Here we have systematically reviewed COVID‐associated arthritis in patients after severe and non‐severe COVID‐19 infection by aggregating both post‐COVID inflammatory and viral arthritides studies.

## 2 METHODS

All procedures used in this systematic review have complied with the preferred reporting items for systematic review guidelines (PRISMA).^9^ The PRISMA flow chart diagram is documented in Supporting Information: S1 File.

### 2.1 Search strategy

To claim the cases, PubMed (MEDLINE/PMC), SCOPUS, EMBASE, and other valid resources, were comprehensively searched by using the following keywords: “Inflammatory Arthritis” OR “Post‐Infectious Arthritis” OR “Reactive Arthritis” OR “Reiter's Syndrome” OR “Sacroiliitis” AND “COVID‐19” OR “SARS‐CoV‐2” OR “Coronavirus Disease‐19.” Additional data are given in Supporting Information: S2 File.

### 2.2 Inclusion criteria

Published articles in English on both IA (including Re‐A, RA, AS, PA, GA, and lupus arthritis) and viral arthritis occurring after COVID‐19 infection; which reported COVID‐19 severity were included. Although numerous papers clearly stated that their patients were diagnosed with severe or non‐severe COVID‐19, some did not. To classify these unidentified patients, intensive care unit admission or hospitalization due to COVID‐19 was considered a positive criterion for COVID‐19 severity.

### 2.3 Exclusion criteria

Cases or articles with undetermined laboratory diagnostic tests (nasopharyngeal/or oropharyngeal PCR swab, antigen test, or serological examination) for COVID‐19 were excluded. Arthralgia was the only complication of some patients; these patients were also excluded.

### 2.4 Data synthesis and quality assessment

We collected the following data for each study: first author and published year, nationality, age and sex, type of arthritis, COVID‐19 severity, number and pattern of involved joints, the basis of COVID diagnosis test, the interval between initiation of COVID‐19 infection and the onset of arthritis, basis of arthritides diagnosis, synovial fluid analysis (presence of germs or crystals), consisting auto‐antibodies rheumatologic antibodies and human leukocyte antigen B27 (HLA‐B27), history of RMDs, history of non‐RMD comorbidities, treatment, outcome, sexually transmitted disease (STD) tests results, extra‐articular manifestations, and history of recent vaccine injection. We summarized the extracted data by classifying the results into two main groups; COVID‐associated arthritis following non‐severe COVID‐19 and COVID‐associated arthritis following severe COVID‐19. The JBI checklist was used to assess the quality of selected studies in parallel by two reviewers (M. Z. and P. A.), then the results were structured in a qualitative synthesis.

## 3 RESULTS

### 3.1 Study characteristics

**Figure 1:**
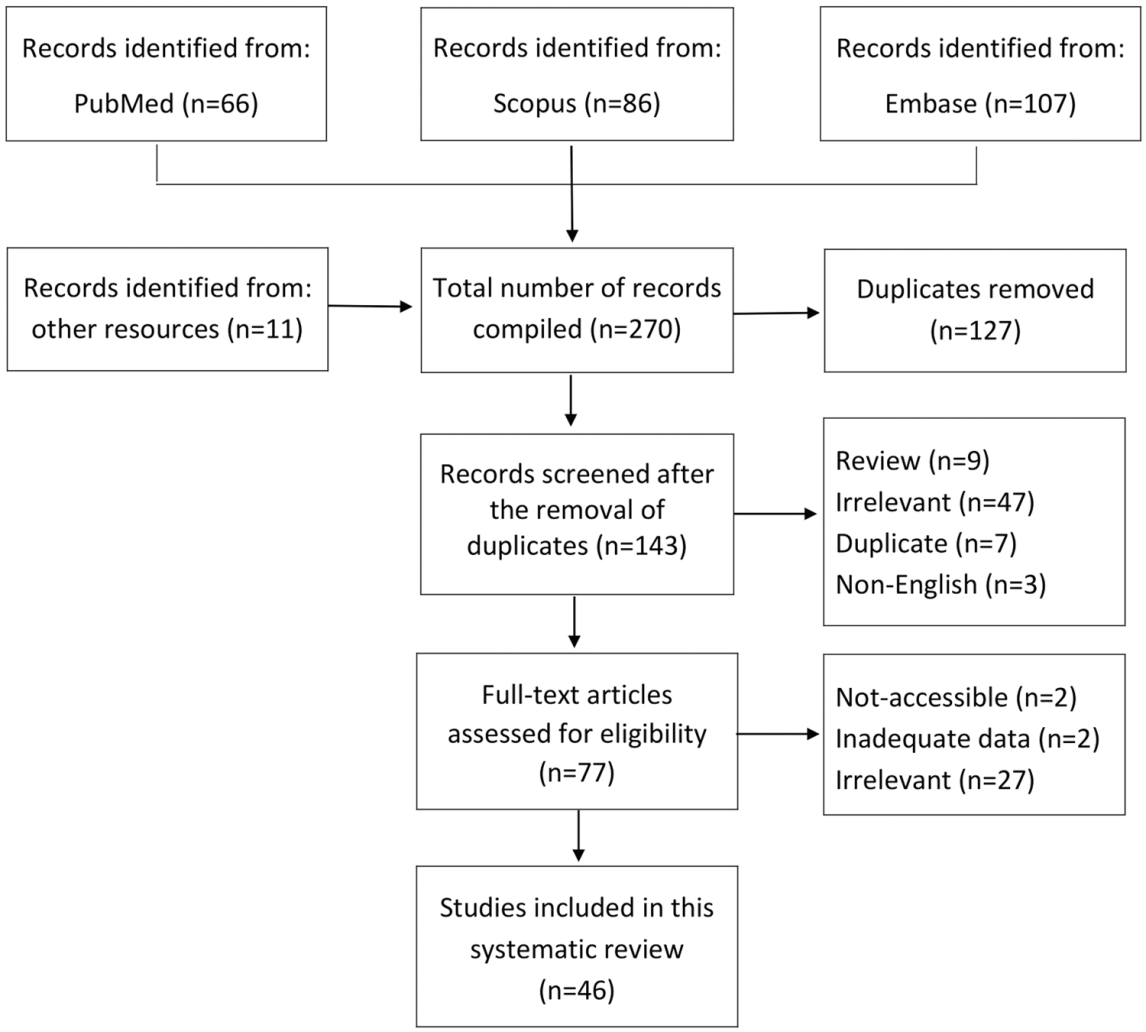
The flow diagram for databases search.

Our search primarily included all published articles in any language until Febuary 20, 2023, and 271 papers were collected. Duplicate reports were initially removed; then, the titles, abstracts, and full texts were separately reviewed by two authors (M. Z. and P. A.). Non‐English, review, and irrelevant articles were excluded. Cases with post‐COVID arthritis, including case reports, case series, letters, editorial papers, comments, and conferences, were included for eligibility assessment, and documents with inadequate clinical data were excluded. Finally, 41 case reports (45 patients, Table 1)^10^, ^11^, ^12^, ^13^, ^14^, ^15^, ^16^, ^17^, ^18^, ^19^, ^20^, ^21^, ^22^, ^23^, ^24^, ^25^, ^26^, ^27^, ^28^, ^29^, ^30^, ^31^, ^32^, ^33^, ^34^, ^35^, ^36^, ^37^, ^38^, ^39^, ^40^, ^41^, ^42^, ^43^, ^44^, ^45^, ^46^, ^47^, ^48^, ^49^, ^50^ and 5 case series (50 patients, Table 2),^51^, ^52^, ^53^, ^54^, ^55^ with a total number of 95 patients (46 studies), were included in this systematic review. The flow diagram for the search of databases is given in Figure 1.

**Table 1:** Case reports.

First author/year	Covid‐19 severity	Type of arthritis	Age/sex	Pattern of joint involvement	Interval between Covid‐19 & arthritis	Basis of arthritis diagnosis	SF culture/crystals	HLA‐B27 antigen	Auto‐antibodies	History of RMDs	Non‐RMD comorbidities	Treatment	Outcome	Extra‐articular manifestations
Danssaert et al. (2020)^10^	Non‐severe	Post‐COVID Re‐A	37 year/F	Extensor tenosynovitis of the right hand (of the second, third, & forth compartments)	12 days after Covid‐19 diagnosis	MRI, ultrasound, & clinical findings	NM/NM	NM	ANA, RF negative	No	CHF, asthma, GERD, obesity, & history of bariatric surgery	Topical NSAID, gabapentin, opioid	Moderately improved	None
Sidhu et al. (2020)^11^	Non‐severe	Post‐COVID Re‐A	31 year/F	Oligoarthritis of the right wrist, right elbow, & both knees	10 days after Covid‐19 symptoms	Clinical findings	NM/NM	Negative	ANA, RF ANCA & anti‐CCP negative	No	Platelet dysfunction	Oral steroid	Markedly improved	Urticarial rashes
De Stefano et al. (2020)^12^	Non‐severe	COVID‐related arthritis	30 s/M	Monoarthritis of the right elbow	26 days after Covid‐19 symptoms & diagnosis	SF analysis, ultrasound, & clinical findings	NM/no	Negative	ANA, ENA, RF & anti‐CCP negative	No	No	Topical steroid & oral NSAID	Improved pain and functional limitation	Psoriatic skin lesions
Jali et al. (2020)^13^	Non‐severe	Post‐COVID Re‐A	39 year/F	Polyarthritis of left second DIP, fifth DIP & right second PIP, third PIP, fifth DIP	3 weeks after Covid‐19 infection	Clinical findings	NM/NM	NM	ANA, RF & anti‐CCP negative	No	No	Oral NSAID	Improved in 2 weeks, no relapse in 2 months	None
Mukarram et al. (2020)^14^	Non‐severe	Post‐COVID Re‐A	34 year/M	Monoarthritis of the right knee	19 days after Covid‐19 symptoms & 13 days after diagnosis	MRI & clinical findings	NM/NM	NM	NM	No	No	Oral NSAID & inta‐articular steroid	Completely resolved in 10 days	None
Gibson et al. (2020)^15^	Non‐severe	Post‐COVID IA	37 year/M	Polyarthritis of both wrists & PIPs	5 weeks after Covid‐19 diagnosis	Clinical findings	NM/NM	NM	ANA, RF & anti‐CCP negative	No	NAFLD	Oral NSAID & oral steroid	Symptomatically improved	None
Saricaoglu et al. (2020)^16^	Severe	Post‐COVID Re‐A	73 year/M	polyarthritis of Left first MTP, PIP, DIP & right second PIP, DIP	22 days after covid‐19 symptoms & 15 days after diagnosis	Clinical findings	NM/NM	NM	RF & anti‐CCP negative	No	DM‐2, HTN, CAD	Oral NSAID	Completely resolved, 12−14 days later	NM
Yokogawa et al. (2020)^17^	Severe	COVID‐related arthritis	57 year/F	Oligoarthritis of the left wrist, right shoulder, & both knees	20 days after Covid‐19 symptoms & 17 days after diagnosis	SF analysis & clinical findings	NM/no	NM	ANA, RF & anti‐CCP negative	No	HTN, DLM	No treatment	Resolved spontaneously in 1 month	NM
Gasparotto et al. (2020)^18^	Severe	COVID‐related arthritis	60 year/M	oligoarthritis of the right knee and right ankle	32 days after Covid‐19 diagnosis	SF analysis, ultrasound & clinical findings	Negative/no	Negative	ANA, RF & anti‐CCP negative	No	No	Oral NSAID	Improved in 3 weeks; no relapse in 6 months	None
Alivernini et al. (2020)^19^	Non‐severe	COVID‐related arthritis	61 year/M	Polyarthritis (NM)	At Covid‐19 symptoms & diagnosis	SF analysis, ST biopsy, ultrasound, & clinical findings	NM/No	NM	ACPA & RF negative	No	NM	Oral baricitinib & oral steroid	Progressively improved	NM
	Severe	RA‐flare up	50 year/F	Polyarthritis (NM)	9 days after Covid‐19 diagnosis & symptoms	SF analysis, ST biopsy, ultrasound, & clinical findings	NM/NM	NM	ACPA & RF positive	RA	NM	Subcutaneous sarilumab	Improved	NM
Shokraee et al. (2021)^20^	Severe	Post‐COVID Re‐A	58 year/F	Monoarthritis of the right hip & sacroiliitis of right sacroiliac joint	15 days after Covid‐19 symptoms	MRI, ultrasound & clinical findings	NM/NM	NM	NM	No	DM‐2, HTN, CHD	Oral NSAID & Intramuscular steroid	Remission after 14 days	NM
Ouedraogo et al. (2021)^21^	Severe (critical)	Post‐COVID Re‐A	45 year/M	Oligoarthritis of both shoulders, left elbow & left knee	48 days after Covid‐19 symptoms	SF analysis & clinical findings	Negative/No	NM	RF & anti‐CCP negative	No	Chronic low back pain status post‐spinal fusion	Oral steroid	Significantly improved	None
Hønge et al. (2021)^22^	Severe	Post‐COVID Re‐A	53 year/F	Polyarthritis of the right knee, both ankles & lateral side of left foot	20 days after Covid‐19 symptoms & diagnosis	SF analysis & clinical findings	Negative/No	Negative	ANA, RF & anti‐CCP negative	No	Overweight (BMI = 26.5 kg/m^2^)	Oral NSAID & oral steroid	Completely recovered 4 months after Covid‐19	NM
Kocyigit et al. (2021)^23^	Severe	Post‐COVID Re‐A	53 year/F	Monoarthritis of the left knee	41 days after Covid‐19 symptoms	SF analysis, ultrasound, & clinical findings	Negative/No	Negative	ANA, RF & anti‐CPA negative	No	HTN	Oral NSAID	Recovered	None
Apaydin et al. (2021)^24^	Non‐severe	Post‐COVID Re‐A	37 year/M	Polyarthritis of both knees, wrists, ankles, elbows, and MTP joints	1 week after Covid‐19 symptoms, at Covid‐19 diagnosis	SF analysis & clinical findings	Negative/NM	Positive	ANA, ENA, ANCA, RF & anti‐CCP negative	No	No	Oral steroid & oral SSZ	Recurred after 3 days, then improved in 1 month	Watery diarrhea
Cincinelli et al. (2021)^25^	Non‐severe	Post‐COVID IA	27 year/M	Monoarthritis of the right first MCP	2 weeks after Covid‐19 symptoms	Clinical findings	NM/NM	NM	NM	Nail psoriasis	No	Oral NSAID & Oral steroid	resolved	None
Colatutto et al. (2021)^26^	Non‐severe	post‐COVID‐ sacroiliitis	53 year/F	Sacroiliitis of bilateral sacro‐iliac joints	Within 1 month after Covid‐19 symptoms	MRI & clinical findings	NM/NM	Negative	HLA‐B8, B57 positive, ANA, RF & anti‐SSA/SSB negative	No	Autoimmune hypothyroidism	Oral NSAID	Recovered	NM
	Non‐severe	Post‐COVID‐ sacroiliitis	58 year/F	Sacroiliitis of bilateral sacro‐iliac joints	Within 1 month after Covid‐19 symptoms	MRI & clinical findings	NM/NM	Negative	HLA‐B8,B57 positive, ANA, RF & anti‐SSA/SSB negative	No	Autoimmune hypothyroidism	Oral NSAID	Recovered	NM
Coath et al. (2021)^27^	Non‐severe	Post‐COVID Re‐A	53 year/M	Sacroiliitis of the bilateral sacroiliac joint, left first costovertebral, & costotransverse	NM	MRI & clinical findings	NM/NM	Positive	NM	No	Cured lumbar disc herniation, DLM	Oral NSAID & intramuscular steroid	Resolved in 3 months	NM
El Hasbani et al. (2021)^28^	Non‐severe	Post‐COVID SA	25 year/M	Oligoarthritis of the left ankle & right elbow, sacroiliitis of the bilateral sacroiliac joint	19 days after Covid‐19 infection	MRI & clinical findings	NM/NM	Positive	ANA, RF & anti‐CCP negative	No	No	Oral NSAID, oral steroid & oral SSZ	Little response to NSAID, recovered after SSZ & steroid in 1 month	None
	Non‐severe	Post‐COVID SA	57 year/M	Monoarthritis of the left wrist	42 days after Covid‐19 diagnosis	MRI & clinical findings	NM/NM	Positive	ANA, ENA, RF & anti‐CCP negative	No	DLM, HTN	Oral NSAID & oral steroid	Completely resolved in a short period	None
Basheikh et al. (2022)^29^	Non‐severe	Post‐COVID Re‐A	43 year/M	Axial (severe back pain)	15 days after Covid‐19 diagnosis	Clinical findings	NM/NM	Negative	ANA & RF negative	No	Obesity (BMI = 34 kg/m^2^)	Oral NSAID, oral steroid, topical steroid	Recovered after 2 months	Balanitis & bilateral conjunctivitis
Dombret et al. (2022)^30^	Non‐severe	Post‐COVID Re‐A	30 year/F	Oligoarthritis of the second left MTP & left ankle	16 days after Covid‐19 diagnosis	SF analysis, ultrasound, & clinical findings	NM/no	Positive	ANA, RF & anti‐CCP negative	No	No	Oral NSAID, intramuscular steroid & oral steroid	Improved	Bilateral conjunctivitis
Shimoyama et al. (2022)^31^	Non‐severe	Post‐COVID Re‐A	37 year/M	Monoarthritis of the right ankle	6 days after Covid‐19 symptoms & diagnosis	MRI, SF analysis, & clinical findings	Negative/no	Negative	ANA, RF & anti‐CCP negative	Gout (right ankle)	Hyperuricemia, history of right ankle fracture & gout attacks	Oral NSAID & inta‐articular steroid	Symptoms persisted	None
Quaytman et al. (2022)^32^	Non‐severe	Post‐COVID Re‐A	48 year/M	Oligoarthritis of both hips & shoulders with enthesitis	NM	MRI & clinical findings	NM/NM	Negative	Anti‐ds DNA, ANA, anti‐Smith, RNP Ab, chromatin Ab, ANCA, & anti SSA/SSB negative	No	Celiac artery compression syndrome & lung adenocarcinoma	Oral NSAID, oral steroid & oral SSZ	Partially resolved	Silent thyroiditis
Ganta et al. (2022)^33^	Non‐severe	Post‐COVID Re‐A	18 year/M	Monoarthritis of the right knee	3 weeks after Covid‐19 symptoms & diagnosis	SF analysis & clinical findings	Negative/no	Negative	ANA, RF & ACPA negative	No	Refractory Hodgkins lymphoma	Oral NSAID	Completely recovered after few days	None
Ruiz‐del‐Valle et al. (2022)^34^	Non‐severe	Post‐COVID Re‐A	19 year/M	Polyarthritis of both knees, ankles, wrists, & small joints	NM	Clinical findings	NM/NM	Negative	Anti‐ds DNA, ANA, RF, ENA, & anti‐CCP negative	No	Alopecia areata & pitytriasis versicolor	Oral steroid	improved	Rash on his back
Luceño et al. (2023)^35^	Non‐severe	Post‐COVID Re‐A	37 year/M	oligoarthritis of the third DIP joint of the hand & first right MTP	At Covid‐19 symptoms & diagnosis	Clinical findings	NM/NM	NM	RF & anti‐CCP negative	Undifferentiated IA 2 years earlier	No	Oral NSAID & oral anti‐histamine	Resolved after 2 to 3 weeks without sequelae	Rashes
Talarico et al. (2020)^36^	Non‐severe	COVID‐related arthritis	45 year/M	symmetric polyarthritis of the MCP & PIP joints of both hands, right wrist	1 week before Covid symptoms & 2 weeks before diagnosis	Clinical findings	NM/NM	NM	RF negative, anti‐CCP positive	No	NM	Oral steroid	Complete remission in 3 months	NM
Parisi et al. (2020)^37^	Non‐severe	Post‐COVID viral Re‐A	58 year/F	monoarthritis of an ankle with tendonitis of the Achilles tendon	25 days after Covid‐19 symptoms	ultrasound & clinical findings	NM/NM	Negative	Anti‐ds DNA, ANA, RF & anti‐CCP negative	No	NM	Oral NSAID	Arthralgia resolved but synovitis remained	Diarrhea
Fragata et al. (2020)^38^	Non‐severe	Post‐COVID Re‐A	41 year/F	polyarthritis of third, forth PIPs, DIPs & first MCPs of both hands	4 weeks after Covid‐19 symptoms	Clinical findings	NM/NM	NM	ANA, RF, ACPA, ENA, & Anti‐ds DNA negative	No	NM	Oral NSAID & oral steroid	Recovered	NM
Houshmand et al. (2020)^39^	Non‐severe	Post‐COVID Re‐A	10 year/M	Oligoarthritis of both knees & right elbow	2 days after Covid‐19 symptoms & at diagnosis	SF analysis & clinical findings	NM/NM (no fluid)	NM	ANA & RF negative	No	NM	Oral anti‐histamines	Improved in 72 h	Urticarial rashes
Salvatierra et al. (2020)^40^	Non‐severe	COVID‐related Re‐A	16 year/F	Dactylitis of toes (second, forth & fifth of left of toes)	3 weeks after Covid‐19 symptoms	Clinical findings	NM/NM	Negative	ANA, RF negative	No	NM	Oral NSAID	Resolved in 5 days	NM
Ono et al. (2020)^41^	Severe (critical)	Post‐COVID Re‐A	50 s/M	Oligoarthritis of both ankles with mild enthesitis in the right Achilles tendon	21 days after Covid‐19 symptoms & 20 days after diagnosis	SF analysis & clinical findings	Negative/no	Negative	ANA, RF & anti‐CCP negative	No	Steatohepatitis	Oral NSAID & inta‐articular steroid	Moderately improved	None
Sureja et al. (2021)^30^	Severe	Post‐COVID Re‐A	27 year/F	polyarthritis of both knees, ankles, mid feet, & small joints of the right hand	2 weeks after Covid‐19 diagnosis	Clinical findings	NM/NM	Negative	ANA & anti‐CPA negative, RF positive	No	NM	Oral NSAID, oral steroid & oral opioid	Significantly improved at 4 weeks follow‐up	None
Santacruz et al. (2021)^42^	Severe	Post‐COVID Re‐A	30 year/F	Dactilytis of the forth toe of the left foot	more than 1 month after Covid‐19 symptoms	Clinical findings	NM/NM	Positive	HLA‐B57 positive	NM	NM	Oral steroid	Partial remission	Bilateral conjunctivitis, psoriatic skin lesions, oral lesions, vulvitis
Di Carlo et al. (2021)^43^	Non‐severe	Post‐COVID Re‐A	55 year/M	Monoarthritis of the right ankle, tenosynovitis of the posterior tibial tendon sheath	37 days after covid‐19 infection	Clinical findings	NM/NM	Negative	NM	No	NM	Oral steroid	Recovered	NM
Dutta et al. (2021)^44^	Non‐severe	Post‐COVID Re‐A	14 year/M	Polyarthritis of the right elbow, both knees & ankles	3 weeks after Covid‐19 diagnosis	Ultrasound & clinical findings	NM/NM	Negative	ANA & anti‐CCP negative	No	NM	Oral NSAID & intravascular steroid	Significantly improved	None
Saikali et al. (2021)^45^	Non‐severe	Post‐COVID sacroiliitis (SA)	21 year/F	Sacroiliitis of bilateral sacro‐iliac joint	NM	MRI & clinical findings	NM/NM	Negative	ANA & RF negative	No	NM	Certolizumab	Impressively improved in 2 weeks	Mild diarrhea
Sinaei et al. (2021)^46^	Non‐severe	Post‐COVID Re‐A	8 year/M	Monoarthritis of the left hip	1 week after Covid‐19 symptoms	MRI & clinical findings	NM/NM	NM	ANA negative, RF positive	No	NM	Oral NSAID	Recovered within a week	NM
	Non‐severe	Post‐COVID Re‐A	6 year/F	Oligoarthritis of bilateral hips	1 week after Covid‐19 symptoms	MRI & clinical findings	NM/NM	NM	ANA & RF negative	No	History of limping & right side hydro‐nephrosis	Oral NSAID	Recovered after 4 days, no relapse in 45 days	NM
Jabalameli et al. (2022)^47^	Non‐severe	Post‐COVID Re‐A	28 year/M	Monoarthritis of the right knee	8 days after Covid‐19 symptoms	SF analysis & clinical findings	Negative/NM	Negative	Anti‐ds DNA, ANA, RF & anti‐CCP negative	No	NM	Oral NSAID	improved	NM
Liew et al. (2020)^48^	Non‐severe	Post‐COVID Re‐A	47 year/M	Monoarthritis of the right knee	At Covid‐19 symptoms & 4 days after diagnosis	SF analysis & clinical findings	Negative/No	NM	NM	No	NM	Oral NSAID & inta‐articular steroid	NM	Balanitis
Waller et al. (2020)^49^	Non‐severe	Post‐COVID Re‐A	16 year/F	Polyarthritis of bilateral MCPs, wrist, shoulder, hip, & knee	14 days after Covid‐19 symptoms	Clinical findings	NM/NM	NM	ANA, RF & ANCA negative	No	NM	NM	Fully resolved after 2 weeks	Rashes

Abbreviations: ACPA, anti‐citrullinated protein autoantibody; NSAID, nonsteroidal anti‐inflammatory drugs; RMDs, rheumatic and musculoskeletal diseases.

**Table 2:** Case series.

First author/year	Covid‐19 severity	Type of arthritis	Age mean (year)	Sex ratio M:F	Patterns of joint involvement	Common location of joint involvement	Interval between Covid‐19 & arthritis	Basis of arthritis diagnosis	SF culture/crystals	HLA‐B27 antigen & other auto‐anti‐bodies	History of RMDs	Non‐RMD comorbidities	Treatment	Outcome	Extra‐articular manifestations
Visalakshy et al. (2022)^51^	Non‐severe: 3/4	Post‐COVID: Re‐A: 2/3 viral arthritis: 1/3	53.67	1:2	Peripheral: 3/3 (mono: 2, oligo: 1, poly: 0)	Knee: 3 Ankle: 1	More than 1.7 weeks after covid‐19 infection	Clinical findings: 3/3	NM	Negative RF, ANA, Anti‐CCP: 3/3	No history of RMD: 3	(Out of 3 patients) HTN: 2 DM‐2: 1 DLP: 1 IDA: 1	Oral NSAID: 2 Colchicine: 1 Oral steroid: 1 IV steroid: 2	Resolved: 3	None
	Severe: 1/4	Undifferentiated arthritis: 1/1	37	1:0	Peripheral: 1/1 (mono: 0, oligo: 1, poly: 0)	Knee: 1 (bilateral: 1/1) Ankle:1	3 months after Covid‐19 infection	Clinical findings: 1/1	NM	Negative RF, ANA, Anti‐CCP: 1/1	No history of RMD: 1	(Out of 1 patient) DM‐2 with nephropathy:1	Oral steroid: 1 HCQ: 1	NM	NM
Sinh et al. (2022)^52^	Non‐severe: 9/9	Post‐COVID Re‐A: 9/9	NM	NM	Peripheral: 8/9 (NM) axial: 1/9 with enthesitis: 3/14	NM	NM	Clinical findings: 9/9 MRI: 1/9 (sacroiliitis)	NM	Positive HLA‐B27: 1/3 Positive RF:2/8	No history of RMD: 9	NM	NM	NM	NM
	Severe: 2/23	Post‐COVID Re‐A: 2/2 (AS:2/2)	49	0:2	Peripheral: 2/2 (mono: 0, oligo: 2, poly: 0) axial: 2/2 with enthesitis: 2/2	Knee: 2 (bilateral: 2/2)	23 days after covid‐19 symptoms	Clinical findings: 2/2	NM	Negative RF, ANA, Anti‐CCP: 2/2	No history of RMD: 2	(Out of 1 patient) Hypothyroidism:1	Oral NSAID: 2	Resolved: 2 Relapse: 0	None
Pal et al. (2023)^53^	Non‐severe: 21/23	Post‐COVID Re‐A: 21/21 (AS: 5/21)	42.24	1:2	Peripheral: 21/21 (mono: 4, oligo: 11, poly: 6) axial: 5/21 with enthesitis:7/21	Knee: 14 (bilateral: 7/14) Ankle: 15, wrist: 7 Small joints: 3 Elbow: 1 Shoulder: 2 (bilateral: 1/2)	26.3 days after Covid‐19 symptoms	Clinical findings: 21/21	NM	Negative RF, ANA, Anti‐CCP: 21/21	No history of RMD: 21	(Out of 4 patients) HTN:4 DM‐2:1	Oral NSAID:19 Oral steroid: 9 IA steroid: 2 MTX: 2 HCQ: 2	Resolved: 21 Relapse: 0	(Out of 2 patients) Rash: 1 Bilateral Conjunctivitis:1
Vogler et al. (2022)^54^	Non‐severe: 8/10	RMD flare‐up: 4/8	60.25	3:1	Peripheral: 4/4 (mono: 1, oligo: 1, poly: 2) with tenosynovitis: 2/4	Wrist:3 (bilateral: 1/3) Small joints: 4	4.5 days after Covid‐19 symptoms& 3.2 days after diagnosis	Ultrasound & clinical findings: 4/4	NM	NM	RA: 2 PA: 1 pSS + SCLE: 1 chondrocalcinosis: 1	(Out of 1 patient) HTN:1 BPH:1 MGUS:1	MTX: 3 SSZ: 1 Adalimumab: 1 Abatacept: 1 Sarilumab: 1	Partially remitted: 4	NM
		Post‐COVID Re‐A: 4/8	51.5	1:3	Peripheral:4/4 (mono: 0, oligo: 1, poly: 3) tenosynovitis: 2/4	Wrist: 3 (bilateral: 1/3) Small joints: 3 Knee: 2 Ankle: 2 Shoulder: 1	10 weeks days after Covid‐19 symptoms & diagnosis	Ultrasound & clinical findings: 4/4	NM	NM	No history of RMD: 4	(Out of 4 patients) DLP: 1 Migrane: 1 Trigger finger: 1 NSCLC: 1	NSAID: 4 steroid: 2 MTX: 1 SSZ: 1	Resolved: 3 Relapse: 1	NM
Vogle et al. (2022)^54^	Severe: 2/10	Post‐COVID Re‐A: 2/2	77.5	1:1	Peripheral: 2/2 (mono: 1, oligo: 1, poly: 0)	Wrist:1 (bilateral: 1/1) Small joints:1	2.5 days after Covid‐19 symptoms & diagnosis	Ultrasound & clinical findings: 2/2	NM	NM	No history of RMD: 2	(Out of 2 patients) HTN:1 DLP:2 IDA:1	steroid: 2	Resolved: 2	NM
Lopez‐Gonzalez et al. (2020)^55^	Non‐severe: 2/4	RMD flare‐up: 2/2	67.5	2:0	Peripheral: 2/2 (mono: 1, oligo: 1, poly: 0)	Knee:1 (bilateral: 1/1) MTP:1	8 days after Covid‐19 symptoms	SF analysis & clinical findings: 2/2	SF culture: 1/2 negative 1/2 ND Crystals: 1/2 MSU 1/2 CPP	NM	Recurrent arthritis: 2 Gout: 1	NM	Colchicine: 1 IA steroid: 2	Resolved: 2	NM

Abbreviations: ANA, antinuclear antibodies; anti‐CCP, anti‐cyclic citrullinated peptide antibodies; AS, ankylosing spondylitis; BPH, benign prostatic hyperplasia; CPP, calcium pyrophosphate; DLP, dyslipidemia; DM‐2, diabetes mellitus‐type2; F, female; HCQ, hydroxychloroquine; HLA‐B27, human leukocyte antigen‐B27; HTN, hypertension; IA, intra‐articular; IDA, iron deficiency anemia; IM, intramuscular; IV, intravascular; M, male; MCP, metacarpophalangeal joint; MGUS, monoclonal gammopathy of undetermined significance; mono, monoarthritis; MSU, monosodium urate; MTP, metatarsophalangeal joint; MTX, methotrexate; ND, not done; NM, not mentioned; NSAID, nonsteroidal anti‐inflammatory drug; NSCLC, non‐small cell lung carcinoma; oligo, oligoarthritis; PA, psoriatic arthritis; poly, polyarthritis; pSS, primary Sjögren's syndrome; Re‐A, reactive arthritis; RF, rheumatoid factor; RMD, rheumatic and musculoskeletal diseases; SCLE, subacute cutaneous lupus erythematosus; SF, synovial fluid; SSZ, sulfasalazine.

**Figure 2:**
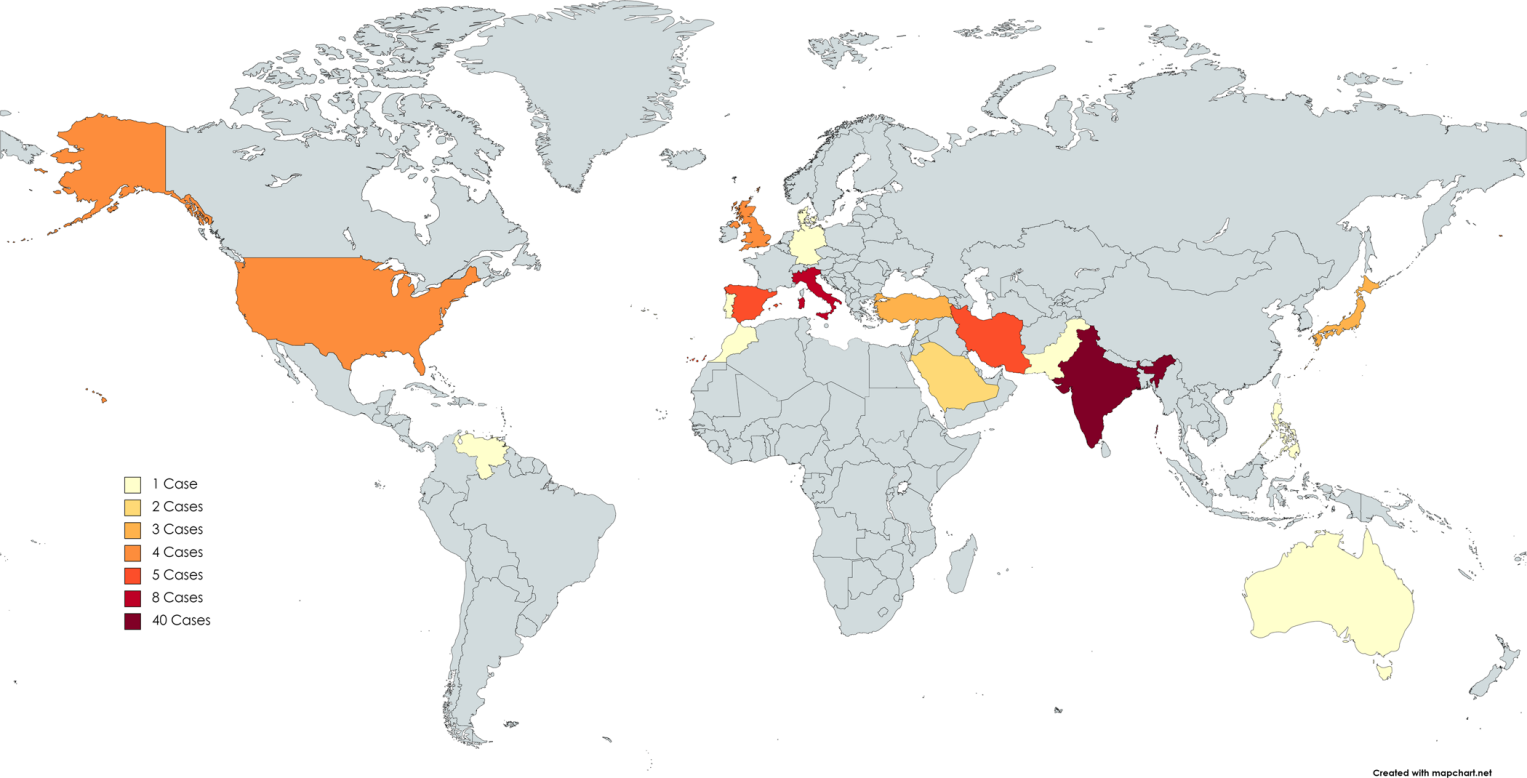
Distribution of COVID‐associated arthritis among 84 reported cases. India: 40 cases; Italy: 8 cases; Spain: 5 cases; Iran: 5 cases; USA: 4 cases; UK: 4 cases; Japan: 3 cases; Turkey: 3 cases; Lebanon: 2 cases; Saudi Arabia: 2 cases; Germany: 1 case; Denmark: 1 case; Australia: 1 case; Pakistan: 1 case; Philippines: 1 case; Portugal: 1 case; Venezuela: 1 case; Morocco: 1 case; not mentioned: 11 cases (10 cases were reported in Europe but with no specific country).

According to the nationalities of reported cases, we made a map chart for the distribution of COVID‐associated arthritis cases worldwide, shown in Figure 2.

### 3.2 Quality of studies

The detailed quality assessment results of the studies are available in Supporting Information: S3 File. Three studies had excellent quality,^51^, ^52^, ^53^ 27 studies had good quality,^10^, ^11^, ^12^, ^13^, ^14^, ^15^, ^16^, ^17^, ^18^, ^19^, ^20^, ^21^, ^22^, ^23^, ^24^, ^25^, ^26^, ^27^, ^28^, ^29^, ^30^, ^31^, ^32^, ^33^, ^34^, ^35^, ^55^ 14 studies had satisfactory quality,^30^, ^36^, ^37^, ^38^, ^39^, ^40^, ^41^, ^42^, ^43^, ^44^, ^45^, ^46^, ^47^, ^48^, ^55^ and two studies had unsatisfactory quality,^49^, ^50^ according to JBI critical appraisal checklist for case reports and case reports.

### 3.3 COVID‐associated arthritis after non‐severe COVID‐19

A total of 77 cases that reported the onset of arthritis after non‐severe COVID‐19 (asymptomatic/mild/moderate) are summarized in Table 1 (case reports) and 2 (case series).^10^, ^11^, ^12^, ^13^, ^14^, ^15^, ^19^, ^24^, ^25^, ^26^, ^27^, ^28^, ^29^, ^30^, ^31^, ^32^, ^33^, ^34^, ^35^, ^36^, ^37^, ^38^, ^39^, ^40^, ^44^, ^45^, ^46^, ^47^, ^48^, ^49^, ^50^, ^51^, ^52^, ^53^, ^54^, ^55^ The patients' COVID infection was diagnosed by nasopharyngeal real‐time polymerase chain reaction (RT‐PCR) or positive immunoglobulin test against SARS‐CoV‐2 by enzyme‐linked immunosorbent assay (ELISA),^15^, ^27^, ^34^, ^40^, ^46^, ^47^, ^53^, ^54^ and the arthritis diagnosis was based on clinical findings in all cases. Patients' sex was noted in 68 cases; 35 were male, and 33 were female. Sixty‐two cases occurred in adults (≥18 years) and 6 cases in juveniles (<18 years); the mean age of 67 reported cases was 41.43 ± 16.45 years; in one article, the exact age was not mentioned, and only the decade of patient age was noted.^12^ In addition, 25 cases declared at least one non‐RMD comorbidity.

While six patients were diagnosed with RMD flare‐ups,^54^, ^55^ 9 cases had a history of RMD; in one article, the patient with a Re‐A diagnosis had a history of gout‐arthritis; however, the author reported this case as a Re‐A.^31^ Also, two patients with a history of nail psoriasis^25^ and undifferentiated IA^35^ were reported as IA and Re‐A, respectively. Other patients experienced their first episode of arthritis by being diagnosed as post‐COVID Re‐A or viral arthritis (58 cases), post‐COVID AS or sacroiliitis (10 cases), COVID‐related arthritis (5 cases), and other IAs (2 cases); these classifications of arthritis types were based on authors' report.

The most prevalent pattern of joint involvement was the peripheral form, reported in 72 patients. Twelve patients experienced axial involvement, and 6 had the peripheral pattern simultaneously. Articular involvement of the peripheral type comprised monoarthritis (in 20 patients), oligoarthritis (in 21 patients), and polyarthritis (in 20 patients). The most frequently involved joints were the knee, reported in 30 cases (bilateral in 14); the ankle, in 27 patients (bilateral in 3); small joints of hands or feet in 21 patients; and wrist, reported in 20 patients; all affected joints are listed in Table 3. Locations and patterns of peripheral arthritis were not mentioned in 10 and 9 patients, respectively.^19^, ^52^ Peri‐articular involvements of peripheral forms, such as enthesitis, tenosynovitis, and tendinitis, were seen in 19 cases, and one of them did not develop arthritis concurrently.^12^ Dactylitis, another peri‐articular manifestation, was noted in 1 patient without arthritis.^40^

**Table 3:** Summary of patients' characteristics and their repetition (and percentage) among reported case.

	The onset of arthritis after non‐severe COVID‐19	The onset of arthritis after severe COVID‐19	*p* Value
Number of total patients	77 (81.05%)	18 (18.95%)	
Adults (≥18 years)	62 (80.52%)	18 (100%)	.191
Juveniles (<18 years)	6 (7.79%)	0 (0%)	
Male	36 (46.75%)	8 (44.44%)	.521
Female	32 (41.56%)	10 (55.56%)	
RMD flare‐up	6 (7.79%)	3 (16.67%)	.247
Non‐RMD arthritis	71 (92.21%)	15 (83.33%)	
History of RMDs	9 (11.69%)	3 (16.67%)	
Non‐RMD comorbidities	25 (32.46%)	10 (55.56%)	
Axial involvement	12 (15.58%)	3 (16.67%)	.211
Peripheral joint involvement	72 (93.50%)	18 (100%)	
❖ Monoarthritis	20 (25.97%)	4 (22.22%)	
❖ Oligoarthritis	21 (27.27%)	9 (50%)	
❖ Polyarthritis	20 (25.97%)	4 (22.22%)	
❖ Dactylitis	1 (1.30%)	1 (5.56%)	
❖ Tenosynovitis, tendinitis, and enthesitis	19 (24.67%)	3 (16.67%)	
Knee joint involvement	30 (38.96%)	10 (55.56%)	.505
Ankle joint involvement	27 (35.06%)	7 (38.89%)	
Small joints involvement	21 (27.27%)	1 (5.56%)	
Wrist joint involvement	20 (25.97%)	2 (11.11%)	
Sacroiliac joint involvement	12 (15.58%)	3 (16.67%)	
Elbow joint involvement	6 (7.79%)	1 (5.56%)	
Shoulder joint involvement	4 (5.19%)	2 (11.11%)	
Hip joint involvement	4 (5.19%)	1 (5.56%)	
Extra‐articular manifestations	15 (19.48%)	1 (5.56%)	.155
Positive HLA‐B27	6 (7.79%)	1 (5.56%)	.442
Positive other autoantibodies	6 (7.79%)	3 (16.67%)	
Positive SF culture	0 (0%)	0 (0%)	
Presence of crystals in SF	2 (2.60%)	2 (11.11%)	
Positive STD tests	0 (0%)	0 (0%)	
History of recent SARS‐CoV‐2 vaccination	2 (2.60%)	0 (0%)	
Early onset of arthritis after COVID‐19 symptoms (≤1 week)	14 (18.18%)	2 (11.11%)	.260
Late onset of arthritis after COVID‐19 symptoms (>1 week)	46 (59.74%)	16 (88.89%)	
No treatment	0 (0%)	1 (5.56%)	.391
NSAIDs	50 (64.94%)	9 (50%)	
Corticosteroids	39 (50.65%)	10 (55.55%)	
DMARDs	13 (16.88%)	1 (5.56%)	
Colchicine	2 (2.60%)	2 (11.11%)	
TNF‐α inhibitors	2 (2.60%)	0 (0%)	
IL‐6 inhibitors	2 (2.60%)	1 (5.56%)	
JAK inhibitors	1 (1.30%)	0 (0%)	
Immunomodulators	1 (1.30%)	0 (0%)	
Anti‐histamines	2 (2.60%)	0 (0%)	
Opioids	1 (1.30%)	1 (5.56%)	
Gabapentin	1 (1.30%)	0 (0%)	
Complete or significant remission	57 (74.03%)	15 (83.33%)	.673
Partial remission	7 (9.09%)	2 (11.11%)	
Relapse or no remission	3 (3.90%)	0 (0%)	

Abbreviations: DMARDs, disease‐modifying anti‐rheumatic drugs; HLA‐B27, human leukocyte antigen B27; IL‐6, interleukin six; JAK, Janus kinase; NSAIDs, nonsteroidal anti‐inflammatory drugs; RMD, rheumatic and musculoskeletal disease; SARS‐CoV‐2, severe acute respiratory syndrome coronavirus 2; SF, synovial fluid; STD, sexually transmitted disease; TNF‐α, tumor necrosis factor‐alpha.

Extra‐articular manifestations like skin rashes,^11^, ^34^, ^35^, ^39^, ^50^, ^53^ conjunctivitis,^29^, ^30^, ^53^ diarrhea,^24^, ^37^, ^46^ balanitis,^29^, ^49^ psoriatic skin lesions,^12^ and silent thyroiditis^32^ were seen in 15 patients; no positive STD test was documented. Additionally, 20 patients underwent the HLA‐B27, and 6 patients had a positive^24^, ^27^, ^28^, ^30^, ^52^; 72 patients had done other rheumatologic auto‐antibodies tests, and 6 patients had a positive result. These positive rheumatologic auto‐antibodies tests include anti‐CCP in 1 patient,^36^ HLA‐B8 and HLA‐B57 in 2 patients,^26^ and RF in 3 patients.^47^, ^52^ The synovial fluid culture was assessed in 6 patients, and none of them was positive; furthermore, synovial fluid analysis for the presence of crystals was performed in 8 cases, and only two samples were positive for calcium pyrophosphate and monosodium urate (MSU) crystals.^55^

The interval between COVID‐19 infection and the onset of arthritis differs from zero days (simultaneous with COVID‐19) to 16 weeks. While the onset of arthritis in 14 cases occurred less than 1 week (≤1 week) after COVID‐19 symptoms,^19^, ^24^, ^31^, ^35^, ^36^, ^39^, ^47^, ^49^, ^51^, ^53^, ^54^ in 46 cases occurred after 1 week^10^, ^11^, ^12^, ^13^, ^14^, ^15^, ^25^, ^26^, ^28^, ^29^, ^30^, ^33^, ^37^, ^38^, ^40^, ^44^, ^45^, ^48^, ^50^, ^51^, ^53^, ^54^, ^55^; this period was not mentioned in 17 patients.

Nonsteroidal anti‐inflammatory drugs (NSAIDs) (in 50 cases) and corticosteroids (in 38 cases) were the most prevalent prescribed drugs for arthritis treatment. Monotherapy with NSAIDs and steroids was used in 20 and 4 patients, respectively. NSAIDs were most commonly used orally; the topical form was used in only one patient.^10^ Corticosteroids were administered via different routes, comprising oral route in 25 patients, intra‐articular route in 5 patients,^14^, ^31^, ^49^, ^53^, ^55^ intravenous route in 3 patients,^45^, ^51^ intramuscular route in 2 patients,^27^, ^30^ and topical form in 2 patients.^12^, ^29^ Prescribed forms of steroids were not listed in 2 cases.^54^ Disease‐modifying antirheumatic drugs (DMARDs), including methotrexate (MTX),^53^, ^54^ sulfasalazine (SSZ),^24^, ^28^, ^32^, ^54^ and hydroxychloroquine (HCQ)^53^ were administered in 13 patients. All prescriptions are listed in Table 3.

Although 57 patients gained complete or significant remission after treatment and follow‐up, 7 patients acquired partial remission, 1 experienced a relapse of symptoms,^54^ and 2 had persistent arthritis with no improvement.^31^, ^37^ Remission status was not mentioned in 10 cases.^49^, ^52^ The history of recent vaccinations against the SARS‐CoV‐2 virus was mentioned in 2 patients.^35^, ^54^

### 3.4 COVID‐associated arthritis after severe COVID‐19

A total of 18 cases that reported the onset of arthritis after severe COVID‐19 (severe/critical) are summarized in Table 1 (case reports) and 2 (case series).^16^, ^17^, ^18^, ^19^, ^20^, ^21^, ^22^, ^23^, ^30^, ^41^, ^43^, ^51^, ^52^, ^54^ Their COVID infection was diagnosed by nasopharyngeal RT‐PCR,^16^, ^17^, ^18^, ^19^, ^20^, ^21^, ^22^, ^23^, ^30^, ^41^, ^51^, ^53^, ^54^ positive antigen test,^43^ or positive IgM against SARS‐CoV‐2 by ELISA,^55^ and the arthritis diagnosis was based on clinical findings in all cases. Eight patients were male, and 10 were female. All cases occurred in adults (≥18 years), and the mean age of 17 patients was 53.05 ± 15.27 years; in one article, the exact age was not mentioned.^41^ Ten cases mentioned the past medical history of non‐RMDs.

While three patients were diagnosed with RMD flare‐ups and had an RMD history,^19^, ^55^ 15 patients developed their first episode of arthritis. The authors reported the classifications of diagnosis as follows: post‐COVID Re‐A (12 cases), post‐COVID AS (2 cases), COVID‐related arthritis (2 cases), and post‐COVID undifferentiated arthritis (1 case).

The prevalent form of joint involvement was the peripheral form reported in 18 patients, and three experienced axial involvement simultaneously. The peripheral form types comprised monoarthritis (in 4 patients), oligoarthritis (in 9 patients), and polyarthritis (in 4 patients). The most frequently involved joints were as follows: the knee, reported in 10 patients (bilateral in 5), and the ankle, in 7 patients (bilateral in 3); All affected joints are listed in Table 3. The location of peripheral arthritis was not mentioned in 1 patient.^19^ Peri‐articular involvements of peripheral forms, such as enthesitis, tenosynovitis, and tendinitis, were seen in 3 cases.^41^, ^53^ Dactylitis is another peri‐articular manifestation noted in 1 patient without arthritis.^43^

Only one patient experienced extra‐articular manifestations, including bilateral conjunctivitis, psoriatic skin lesions, oral lesions, and vulvitis,^43^ and no positive STD test was documented. In addition, 1 patient out of 6 was HLA‐B27 positive^43^; 3 patients out of 17 were positive for other rheumatologic auto‐antibodies, including RF in 2 patients,^19^, ^30^ HLA‐B57 in 1 patient,^43^ and anti‐citrullinated protein autoantibody in 1 patient.^19^ Synovial fluid culture and analysis of crystals were performed in 7 and 8 cases, respectively, and just two samples were positive for MSU crystals.^55^

The interval between COVID‐19 symptoms and the onset of arthritis differs from zero days (simultaneous with COVID‐19) to 3 months. The onset of arthritis in 2 cases occurred less than 1 week (≤1 week) after COVID infection,^54^ and 16 happened after 1 week.^16^, ^17^, ^18^, ^19^, ^20^, ^21^, ^22^, ^23^, ^30^, ^41^, ^43^, ^51^, ^53^, ^55^

Corticosteroids (in 10 cases) and NSAIDs (in 9 cases) were the most prevalent prescribed drugs for arthritis treatment. Monotherapy with steroids and NSAIDs was used in 4 and 5 patients, respectively. Corticosteroids were administered in different types comprising oral route in 6 patients,^21^, ^22^, ^30^, ^43^, ^51^, ^55^ intra‐articular route in 1 patient,^41^ and intramuscular route in 1 patient.^20^ Prescribed forms of steroids were not noted in 2 cases.^54^ HCQ as a kind of DMARDs was administered in 1 case.^51^ All prescribed drugs are listed in Table 3, and 1 patient's arthritis subsided without treatment.^17^ Although 15 patients gained complete or significant remission after treatment or follow‐up, 2 patients acquired partial symptom improvement^41^, ^54^; remission status was not mentioned in 1 case.^51^

**Figure 3:**
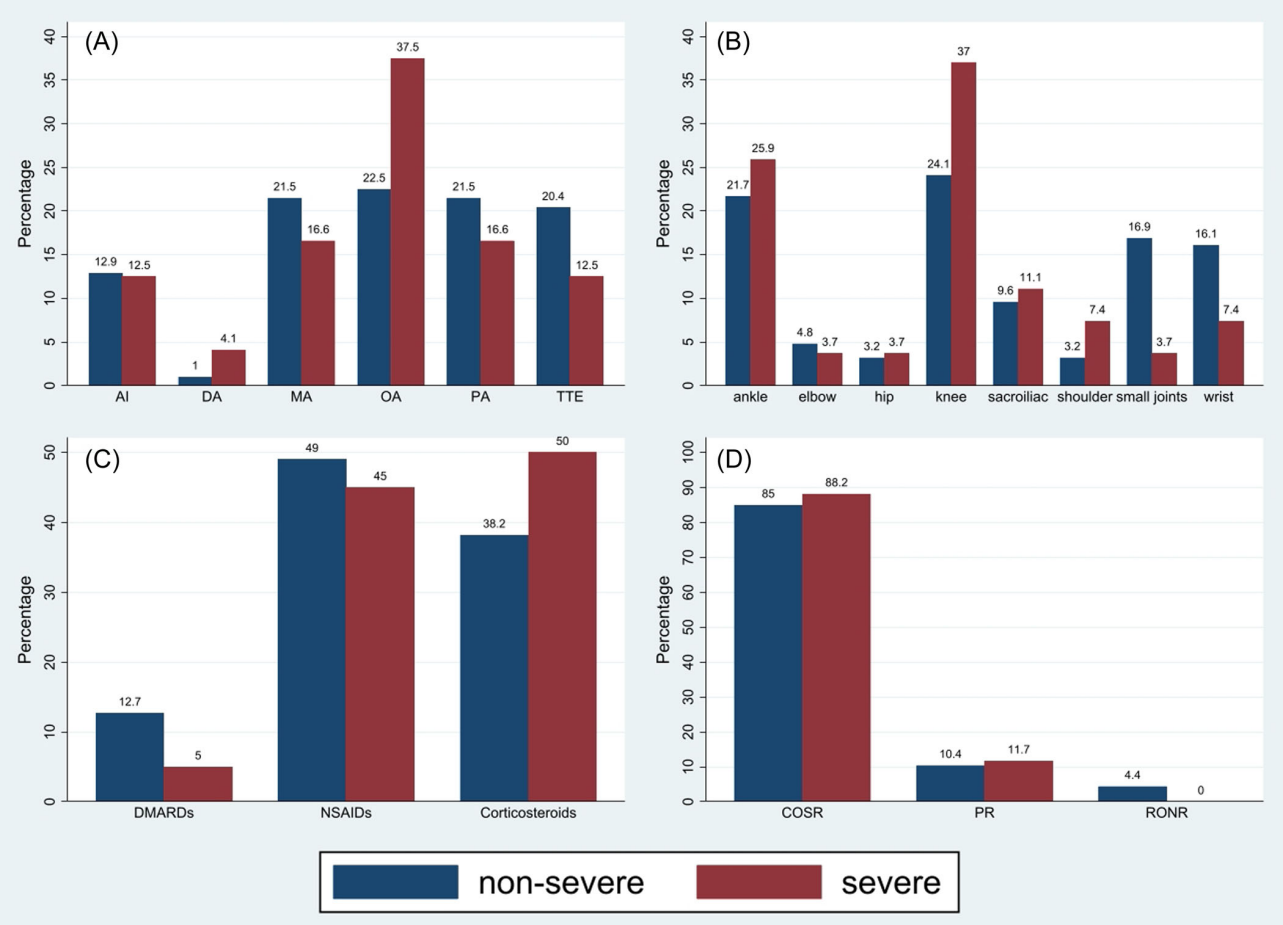
Comparison of the pattern of joint involvements (A), location of involved joints (B), treatments (C), and outcomes (D) between two groups, based on percentage. AI, axial involvement; COSR, complete or significant remission; DA, dactylitis; DMARDs, disease‐modifying anti‐rheumatic drugs; MA, monoarthritis; NSAIDs, nonsteroidal anti‐inflammatory drugs; OA, oligoarthritis; PA, polyarthritis; PR, partial remission; RONR, relapse or no remission; TTE, tenosynovitis, tendinitis, and enthesitis.

Patients' characteristics in both severe and non‐severe groups are summarized in Table 3 and Figure 3. The association between age, sex, type of arthritis, the pattern of joint involvement, location of involved joints, extra‐articular manifestation, lab tests, the onset of arthritis, treatment, outcome, and designated severe and non‐severe groups were assessed using *χ* ^2^ tests (Table 3). No association was determined (*p* > .05).

## 4 DISCUSSION

Re‐A, RA, AS, PA, and GA are common subgroups of IA that frequently arose after COVID‐19 infection. Re‐A and AS were repeatedly reported after infection with viruses such as SARS‐CoV‐2 and were presented as the first episode of arthritis in patients without RMD history. Re‐A often occurs with asymmetric oligoarthritis of the lower limbs, especially the knee joint; AS is mainly copresent with axial involvement, called sacroiliitis; both AS and sacroiliitis are considered subgroups of spondyloarthropathies, that are associated with the HLA‐B27 genetic marker.^56^ RA, PA, and GA are other chronic IA that can be flared up or first appear after viruses like SARS‐CoV‐2.^57^ Although these cases were reported during the coronavirus disease pandemic, other etiologies cannot be entirely excluded.

In this systematic review, we compiled all published data on patients with COVID‐related arthritis. We summarized 95 included patients in two categories: COVID‐associated arthritis following non‐severe COVID‐19 and COVID‐associated arthritis following severe COVID‐19 (Table 3). We used “COVID‐associated arthritis” because there were no definite diagnostic classifications for arthritides after COVID‐19, and the arthritides types were based on the author's point of view. Previously, Farisogullari et al. used the “COVID‐associated arthritis” term instead of both Re‐A and viral arthritis^8^; However, we used this term to contain all common types of IA and viral arthritis.

Following our computation, 81.05% of the patients experienced asymptomatic, mild, or moderate COVID‐19, and 18.95% underwent severe or critical COVID‐19 infection, similar to the COVID‐19 severity rate (19%).^58^ However, the mortality rates are 54.64% for severe COVID‐19 and 5% for non‐severe COVID‐19 infection,^59^ which can mean most severe COVID‐19 cases expired before developing arthritis, and the actual rate of arthritis after severe COVID‐19 could be doubled; consequently, we predict that COVID‐severity may be a risk factor for the occurrence of post‐COVID‐arthritis.

All patients in the severe COVID‐19 group were adults with a mean age of 53.05 ± 15.27 years, and 55.56% of them had at least one non‐RMD comorbidity; on the other hand, in the non‐severe COVID‐19 group, 7.79% of cases were juveniles, and the mean age was 41.43 ± 16.45 years; and 32.46% mentioned at least one non‐RMD comorbidity; which shows the fact that age and comorbidity are the most potent risk factors for severe COVID‐19 outcomes.^60^

According to the authors' reports, 92.21% of patients in the non‐severe COVID‐19 group were diagnosed with non‐RMD arthritis and 7.79% with RMD flare‐ups; however, 11.69% of them declared a history of RMDs and 7.79% had positive rheumatic autoantibodies. We included all COVID‐related arthritides, not solely Re‐A, to avoid missing any related data or cases. In the severe COVID‐19 group, 16.67% of patients were diagnosed with RMD flare‐ups and reported a history of RMDs with positive rheumatic autoantibodies; the rest experienced their first episode of IA. Despite the fact that there were no significant differences in HLA‐B27 positivity between the two groups and most of the positive HLA‐B27 patients were cases with sacroiliitis or cases of non‐axial Re‐As.^56^

Furthermore, two patients who experienced arthritis after non‐severe COVID‐19 declared a history of recent vaccine injections. One of them reported a SARS‐CoV‐2 vaccine (Biontech/Pfizer) injection 9 days before arthritis, whereas the onset of COVID‐infection occurred 8 weeks before arthritis,^54^ so we think it was post‐COVID‐vaccine arthritis rather than post‐COVID‐arthritis, and this situation was numerously reported, before.^3^ In the second case, a SARS‐CoV‐2 vaccine (inactivated Sinovac) was injected 2 months before arthritis and COVID‐19 symptoms^35^; in this case, both COVID‐19 and COVID‐vaccine can be the triggers of arthritis.

While in the non‐severe COVID‐19 group, common peripheral joint involvement patterns consist of oligoarthritis (27.27%), monoarthritis (25.97%), and polyarthritis (25.97%), in the severe COVID‐19 group, the proportion of each pattern was different, oligoarthritis (50%) was the most prevalent, monoarthritis (22.22%), and polyarthritis (22.22%) were following ones (Figure 3); we assume that current distribution of patterns in the non‐severe group was because of the unmentioned cases (11.53%) that could change the oligoarthritis pattern percentage in this group. The knee was the most affected joint in both groups, perhaps due to the high number of Re‐A among the cases^56^ (Figure 3).

As the treatments of arthritis were based on age, comorbidities, RMDs history, arthritis severity, and other personal conditions, we cannot precisely define the best treatment; nevertheless, in both groups, polytherapy (or combination therapy) was more common than monotherapy, and it often included oral NSAIDs with different types of corticosteroids (Figure 3). More aggressive treatments such as DMARDs, TNF‐α inhibitors, immunomodulators, and JAK inhibitors were administered more frequently in the non‐severe COVID‐19 group. However, the complete or significant remission rate was higher in the severe COVID‐19 group (74.03% in the non‐severe and 83.33% in the severe group), and relapse or no remission rate was lower in the severe group (3.90% in the non‐severe and 0% in severe) (Figure 3). The late onset (>1 week) of arthritis after COVID‐19 symptoms in non‐severe and severe COVID‐19 were 59.74% and 88.89%, respectively. Overall, even though the patients in the severe COVID‐19 group developed more serious COVID‐19 symptoms, they experienced milder arthritis with better outcomes and more delayed onsets that required less aggressive therapy; therefore, we suppose that weaker immunity situation in the severe COVID‐19 group, due to aggressive corticosteroids therapy or other aggressive immunosuppressant treatments during hospitalization for COVID‐19 infection, causes increased immune‐mediated complications following COVID‐19. The pathogenesis of post‐viral IA is partially understood. However, one of the hypothetical mechanisms mediating the activation of the inflammatory process is molecular mimicry, which is supposed to be responsible for evoking autoimmune responses in susceptible individuals.^4^, ^18^

Many reviews have been conducted to collect COVID‐related arthritis before,^3^, ^7^, ^8^, ^23^, ^26^, ^32^ but there are some differences between this comprehensive review and them. As mentioned, we collected all IA types following COVID‐19, but others only gathered Re‐A or viral arthritis. We reviewed the case series and the case reports altogether, with a total number of 95 cases. In contrast, others did not review any case series and included fewer patients (at most 33 cases^8^). In addition, we listed data in two different non‐severe and severe COVID‐19 categories to compare them and detect the probable relations between COVID‐infection severity and post‐COVID arthritis severity, which was novel.

Besides all the new data and evaluations, our study had some limitations too; for example, no observational study was done, there were a lot of unmentioned data in some papers, and some cases were better documented than others, leading to variations in the quality of papers. To decrease the risk of bias and improve the quality of evaluations, we listed the case reports and the case series in two separate tables (Tables 1 and 2).

## 5 CONCLUSION

This study compares COVID‐associated arthritis in two non‐severe COVID‐19 and severe COVID‐19 categories by collecting data from 95 cases. We conclude that the prevalence of COVID‐associated arthritis may increase with COVID‐19 severity. However, there is an inverse relationship between COVID‐19 severity and arthritis severity, probably because of weaker immunity conditions following immunosuppressant therapy in patients with severe COVID‐19. We suggest that all non‐severe COVID‐19 patients, even asymptomatic ones, need nonaggressive immunosuppressant treatments (during COVID‐19 infection) to alleviate the immune‐based complications, specifically IA.

## AUTHOR CONTRIBUTIONS

Mahsa Zarpoosh contributed to searching databases, collecting data, and writing the manuscript. Parsa Amirian contributed to searching databases, collecting data, and revision the manuscript.

## CONFLICT OF INTEREST STATEMENT

The authors declare no conflict of interest.

## Supporting information

Supporting information.

Supporting information.

Supporting information.

## Data Availability

The data supporting the present study's findings are available from the corresponding author upon request.

## References

[1] Tan M , Liu Y , Zhou R , et al. Immunopathological characteristics of coronavirus disease 2019 cases in Guangzhou, China. Immunology. 2020;160(3):261‐268. 10.1111/imm.13223 32460357 PMC7283723

[2] Ciaffi J , Meliconi R , Ruscitti P , Berardicurti O , Giacomelli R , Ursini F . Rheumatic manifestations of COVID‐19: a systematic review and meta‐analysis. BMC Rheumatol. 2020;4(1):65. 10.1186/s41927-020-00165-0 33123675 PMC7591274

[3] Bekaryssova D , Yessirkepov M , Zimba O , Gasparyan AY , Ahmed S . Reactive arthritis before and after the onset of the COVID‐19 pandemic. Clin Rheumatol. 2022;41(6):1641‐1652. 10.1007/s10067-022-06120-3 35247132 PMC8898028

[4] Cusick MF , Libbey JE , Fujinami RS . Molecular mimicry as a mechanism of autoimmune disease. Clin Rev Allergy Immunol. 2012;42:102‐111. 10.1007/s12016-011-8294-7 22095454 PMC3266166

[5] Pecani A , Tula J , Karaulli L , Islamaj A , Bala S . AB1092 inflammatory arthritides in post‐COVID‐19 patients: an Albanian experience. Ann Rheum Dis. 2022;81:1664.2. 10.1136/annrheumdis-2022-eular.728

[6] Varache S , Narbonne V , Jousse‐Joulin S , et al. Is routine viral screening useful in patients with recent‐onset polyarthritis of a duration of at least 6 weeks? Results from a nationwide longitudinal prospective cohort study. Arthritis Care Res. 2011;63(11):1565‐1570. 10.1002/acr.20576 21954118

[7] Slouma M , Abbes M , Mehmli T , et al. Reactive arthritis occurring after COVID‐19 infection: a narrative review. Infection. 2023;51(1):37‐45. 10.1007/s15010-022-01858-z 35655110 PMC9162104

[8] Farisogullari B , Pinto AS , Machado PM . COVID‐19‐associated arthritis: an emerging new entity? RMD Open. 2022;8(2):e002026. 10.1136/rmdopen-2021-002026 36100294 PMC9471208

[9] Shamseer L , Moher D , Clarke M , et al. Preferred reporting items for systematic review and meta‐analysis protocols (PRISMA‐P) 2015: elaboration and explanation. BMJ. 2015;349:g7647. 10.1136/bmj.g7647 25555855

[10] Danssaert Z , Raum G , Hemtasilpa S . Reactive arthritis in a 37‐year‐old female with SARS‐CoV2 infection. Cureus. 2020;12(8):9698. 10.7759/cureus.9698 PMC7486113 32923288

[11] Sidhu A , Selvan S , Alkutobi Z . EP02 a rare case of reactive arthritis secondary to COVID‐19. Rheumatol Advan Practice. 2020;4(suppl ment_1):rkaa052‐001. 10.1093/rap/rkaa052.001

[12] De Stefano L , Rossi S , Montecucco C , Bugatti S . Transient monoarthritis and psoriatic skin lesions following COVID‐19. Ann Rheum Dis. 2023;82(4):e86. 10.1136/annrheumdis-2020-218520 32753423

[13] Jali I . Reactive arthritis after COVID‐19 infection. Cureus. 2020;12(11):11761. 10.7759/cureus.11761 PMC7779130 33409010

[14] Mukarram IG , Mukarram MS , Ishaq K , Riaz SU . Post‐COVID‐19 reactive arthritis: an emerging existence in the spectrum of musculoskeletal complications of SARS‐CoV‐2 infection. J Clin Stud Med Case Rep. 2020;7(101):2. 10.24966/CSMC-8801/100101

[15] Gibson M , Sampat K , Coakley G . EP15 A self‐limiting symmetrical polyarthritis following COVID‐19 infection. Rheumatol Advan Practice. 2020;4(suppl ment_1):rkaa052‐014. 10.1093/rap/rkaa052.014

[16] Saricaoglu EM , Hasanoglu I , Guner R . The first reactive arthritis case associated with COVID‐19. J Med Virol. 2021;93(1):192‐193. 10.1002/jmv.26296 32652541 PMC7405389

[17] Yokogawa N , Minematsu N , Katano H , Suzuki T . Case of acute arthritis following SARS‐CoV‐2 infection. Ann Rheum Dis. 2021;80(6):e101. 10.1136/annrheumdis-2020-218281 32591356

[18] Gasparotto M , Framba V , Piovella C , Doria A , Iaccarino L . Post‐COVID‐19 arthritis: a case report and literature review. Clin Rheumatol. 2021;40:3357‐3362. 10.1007/s10067-020-05550-1 33587197 PMC7882861

[19] Alivernini S , Cingolani A , Gessi M , et al. Comparative analysis of synovial inflammation after SARS‐CoV‐2 infection. Ann Rheum Dis. 2021;80(6):e91. 10.1136/annrheumdis-2020-218315 32632032

[20] Shokraee K , Moradi S , Eftekhari T , Shajari R , Masoumi M . Reactive arthritis in the right hip following COVID‐19 infection: a case report. Trop Dis Travel Med Vaccines. 2021;7(1):18. 10.1186/s40794-021-00142-6 34130744 PMC8204059

[21] Ouedraogo F , Navara R , Thapa R , Patel KG . Reactive arthritis post‐SARS‐CoV‐2. Cureus. 2021;13(9):18139. 10.7759/cureus.18139 PMC8526086 34692347

[22] Hønge BL , Hermansen MLF , Storgaard M . Reactive arthritis after COVID‐19. BMJ Case Rep. 2021;14(3):e241375. 10.1136/bcr-2020-241375 PMC7929821 33653867

[23] Kocyigit BF , Akyol A . Reactive arthritis after COVID‐19: a case‐based review. Rheumatol Int. 2021;41(11):2031‐2039. 10.1007/s00296-021-04998-x 34550429 PMC8456072

[24] Apaydin H , Guven SC , Kucuksahin O , Omma A , Erten S . A case of human leukocyte antigen B27 positive reactive arthritis associated with severe acute respiratory syndrome coronavirus 2 infection. North Clin Istanb. 2021;8:423‐424. 10.14744/nci.2020.88965 34585082 PMC8430365

[25] Cincinelli G , Di Taranto R , Orsini F , Rindone A , Murgo A , Caporali R . A case report of monoarthritis in a COVID‐19 patient and literature review: simple actions for complex times. Medicine. 2021;100(23):e26089. 10.1097/md.0000000000026089 34114992 PMC8202614

[26] Colatutto D , Sonaglia A , Zabotti A , Cereser L , Girometti R , Quartuccio L . Post‐COVID‐19 arthritis and sacroiliitis: natural history with longitudinal magnetic resonance imaging study in two cases and review of the literature. Viruses. 2021;13(8):1558. 10.3390/v13081558 34452422 PMC8402767

[27] Coath FL , Mackay J , Gaffney JK . Axial presentation of reactive arthritis secondary to COVID‐19 infection. Rheumatology. 2021;60(7):e232‐e233. 10.1093/rheumatology/keab009 33471106 PMC7928576

[28] El Hasbani G , Jawad A , Uthman I . Axial and peripheral spondyloarthritis triggered by sars‐cov‐2 infection: a report of two cases. Reumatismo. 2021;73(1):59‐63. 10.4081/reumatismo.2021.1374 33874649

[29] Basheikh M . Reactive arthritis after COVID‐19: a case report. Cureus. 2022;14(4):24096. 10.7759/cureus.24096 PMC9106556 35573487

[30] Dombret S , Skapenko A , Schulze‐Koops H . Reactive arthritis after SARS‐CoV‐2 infection. Rmd Open. 2022;8(2):e002519. 36096524 10.1136/rmdopen-2022-002519 PMC9471204

[31] Shimoyama K , Teramoto A , Murahashi Y , et al. Surgically treated reactive arthritis of the ankle after COVID‐19 infection: a case report. J Infect Chemother. 2022;28(4):587‐590. 10.1016/j.jiac.2021.12.028 35016827 PMC8720533

[32] Quaytman J , Gollamudi U , Bass N , Suresh S . Reactive arthritis and silent thyroiditis following SARS‐CoV‐2 infection: case report and review of the literature. Clin Case Rep. 2022;10(2):e05430. 10.1002/ccr3.5430 35154733 PMC8819636

[33] Ganta SR , Garg A , Shah K , et al. Post‐Covid‐19 reactive arthritis in two stem cell transplant recipients. Indian J Hematol Blood Transfusion. 2023;39(1):154‐155. 10.1007/s12288-022-01549-7 PMC9868200 36699433

[34] Ruiz‐del‐Valle V , Sarabia de Ardanaz L , Navidad‐Fuentes M , Martín‐Martín I , Lobato‐Cano R . Reactive arthritis with SARS‐CoV‐2 as a trigger. Reumatología Clínica (English Edition). 2022;18(8):490‐492. 10.1016/j.reumae.2021.11.002 PMC9091329 35562296

[35] Luceno V , Navarra S . Seronegative oligo‐arthritis following covid‐19 infection: a case report. Int J Rheumatic Diseases. 2023;26(S1):216. 10.1111/1756-185X.14505

[36] Talarico R , Stagnaro C , Ferro F , Carli L , Mosca M . Symmetric peripheral polyarthritis developed during SARS‐CoV‐2 infection. Lancet Rheumatol. 2020;2(9):e518‐e519. 10.1016/S2665-9913(20)30216-2 32838313 PMC7357970

[37] Parisi S , Borrelli R , Bianchi S , Fusaro E . Viral arthritis and COVID‐19. Lancet Rheumatol. 2020;2(11):e655‐e657. 10.1016/S2665-9913(20)30348-9 33043303 PMC7535796

[38] AF M . Coronavirus disease 19 (COVID‐19) complicated with post‐viral arthritis. Acta Reumatol Port. 2020;45(4). https://www.arprheumatology.com/section.php?id=1478 33420769

[39] Houshmand H , Abounoori M , Ghaemi R , Bayat S , Houshmand G . Ten‐year‐old boy with atypical COVID‐19 symptom presentation: a case report. Clin Case Rep. 2021;9(1):304‐308. 10.1002/ccr3.3521 33362924 PMC7753279

[40] Salvatierra J , Martínez‐Peñalver D , Salvatierra‐Velasco L . Covid‐19 related dactyitis. Joint Bone Spine. 2020;87(6):660. 10.1016/j.jbspin.2020.06.009 32622040 PMC7328572

[41] Ono K , Kishimoto M , Shimasaki T , et al. Reactive arthritis after COVID‐19 infection. RMD Open. 2020;6(2):e001350. 10.1136/rmdopen-2020-001350 32763956 PMC7722270

[42] Sureja NP , Nandamuri D . Reactive arthritis after SARS‐CoV‐2 infection. Rheumatol Advan Practice. 2021;5(1):rkab001. 10.1093/rap/rkab001 PMC7882147 33615130

[43] Santacruz JC , Londoño J , Santos AM , Arzuaga A , Mantilla MJ . Extra‐articular manifestations in reactive arthritis due to COVID‐19. Cureus. 2021;13(10):18620. 10.7759/cureus.18620 PMC8574203 34765373

[44] Di Carlo M , Tardella M , Salaffi F . Can SARS‐CoV‐2 induce reactive arthritis? Clin Exp Rheumatol. 2021;39:25‐26. 33506755

[45] Dutta S , Dey S , Poddar A , Pal P . Post‐COVID reactive arthritis. Indian J Pediatr. 2022;89:103. 10.1007/s12098-021-03992-2 34687437

[46] Saikali W , Gharib S . The first non‐radiographic axial spondyloarthrits with COVID‐19. Immun Inflamm Dis. 2021;9(3):628‐631. 10.1002/iid3.448 33979033 PMC8239938

[47] Sinaei R , Pezeshki S , Parvaresh S , et al. Post SARS‐CoV‐2 infection reactive arthritis: a brief report of two pediatric cases. Pediatric Rheumatol. 2021;19(1):89. 10.1186/s12969-021-00555-9 PMC8196291 34118941

[48] Jabalameli M , Rahbar M , Karimi Haris H , et al. Misdiagnosis of reactive arthritis with septic arthritis in a coronavirus disease 2019 (COVID‐19)‐positive patient: a case report. Curr Orthopaedic Practice. 2022;33(3):299‐301. 10.1097/BCO.0000000000001111

[49] Liew IY , Mak TM , Cui L , Vasoo S , Lim XR . A case of reactive arthritis secondary to coronavirus disease 2019 infection. JCR: J Clin Rheumatol. 2020;26:233. 10.1097/RHU.0000000000001560 32694352 PMC7437408

[50] Waller R , Price E , Carty S , Ahmed A , Collins D . EP01 post‐COVID‐19 reactive arthritis. Rheumatol Advan Practice. 2020;4(suppl ment_1):rkaa052. 10.1093/rap/rkaa052

[51] Visalakshy J , George T , Easwar SV . Joint manifestations following COVID‐19 infection—a case series of six patients. J Clin Diagnostic Res. 2022;16(4). 10.7860/JCDR/2022/51552.16192

[52] Sinha D , Mondal S , Ghosh A . Coronavirus disease‐19 associated arthritis—an observational study. Indian J Rheumatol. 2022;17(2):153‐156. 10.4103/injr.injr_133_21

[53] Pal A , Roongta R , Mondal S , et al. Does post‐COVID reactive arthritis exist? Experience of a tertiary care centre with a review of the literature. Reumatología Clínica (English Edition). 2023;19(2):67‐73. 10.1016/j.reumae.2022.03.005 PMC9096625 35578636

[54] Vogler D , Ruscitti P , Navarini L , et al. Diagnosis of COVID‐19 associated arthritis in patients with or without underlying rheumatic and musculoskeletal disease supported by musculoskeletal ultrasound: a case series from three European centres. Clin Exp Rheumatol. 2023;41:656‐666. 10.55563/clinexprheumatol/an1yrh 35916289

[55] López‐González MC , Peral‐Garrido ML , Calabuig I , et al. Case series of acute arthritis during COVID‐19 admission. Ann Rheum Dis. 2021;80(4):e58. 10.1136/annrheumdis-2020-217914 32471899

[56] Leirisalo‐Repo M . Reactive spondyloarthritis: epidemiology, clinical features, and treatment. In Ankylosing Spondylitis and the Spondyloarthropathies. Mosby; 2006:53‐64.

[57] Derksen VFAM , Kissel T , Lamers‐Karnebeek FBG , et al. Onset of rheumatoid arthritis after COVID‐19: coincidence or connected? Ann Rheum Dis. 2021;80(8):1096‐1098. 10.1136/annrheumdis-2021-219859 33648960

[58] McIntosh K , Hirsch MS , Bloom A . COVID‐19: clinical features. UpToDate. Post‐TW (ed): UpToDate. 2021.

[59] Mahendra M , Nuchin A , Kumar R , Shreedhar S , Mahesh PA . Predictors of mortality in patients with severe COVID‐19 pneumonia—a retrospective study. Advan Resp Med. 2021;89(2):135‐144. 10.5603/ARM.a2021.0036 33966261

[60] Romero Starke K , Reissig D , Petereit‐Haack G , Schmauder S , Nienhaus A , & Seidler A (2021). The isolated effect of age on the risk of COVID‐19 severe outcomes: a systematic review with meta‐analysis. BMJ Glob Health, 6(12), e006434. 10.1136/bmjgh-2021-006434 PMC8678541 34916273

