# Supplementary material for: COVID‐associated arthritis after severe and non‐severe COVID‐19: A systematic review
Source: Immun Inflamm Dis. 2023 Oct 11;11(10):e1035. doi: 10.1002/iid3.1035 (PMC10566449; doi:10.1002/iid3.1035)
Supplement: Supplementary file 2 — Supporting information. [file IID3-11-e1035-s001.docx]

**COVID-associated arthritis after severe and non-severe COVID-19: a systematic review**

Mahsa Zarpoosh, Parsa Amirian*

**corresponding author; Email:* [*parsapj@gmail.com*](mailto:parsapj@gmail.com)

*General Practitioner, Kermanshah University of Medical Science (KUMS), Kermanshah, Iran*

| **Database** | **Search term** |
| --- | --- |
| Pubmed  (n=66) | ("Arthritis, Reactive"[mesh] OR "Arthritis, Reactive"[tiab] OR "Reactive Arthritides"[tiab] OR "Reactive Arthritis"[tiab] OR "Post-Infectious Arthritides"[tiab] OR "Post-Infectious Arthritis"[tiab] OR "Post Infectious Arthritis"[tiab] OR "Postinfectious Arthritis"[tiab] OR "Postinfectious Arthritides"[tiab] OR "Reiter Syndrome"[tiab] OR "Reiter's Disease"[tiab] OR "Reiters Disease"[tiab] OR "Reiter's Syndrome"[tiab] OR "Reiters Syndrome"[tiab] OR "Reiter Disease"[tiab]) AND ("covid-19"[mesh] OR "covid-19"[tiab] OR "COVID 19"[tiab] OR "SARS CoV 2 Infection"[tiab] OR "2019 Novel Coronavirus Disease"[tiab] OR "2019 Novel Coronavirus Infection"[tiab] OR "2019 nCoV Disease"[tiab] OR "COVID 19 Virus Infection"[tiab] OR "Coronavirus Disease 2019"[tiab] OR "Coronavirus Disease 19"[tiab] OR "Severe Acute Respiratory Syndrome Coronavirus 2 Infection"[tiab] OR "SARS Coronavirus 2 Infection"[tiab] OR "COVID 19 Virus Disease"[tiab] OR "2019 nCoV Infection"[tiab] OR "COVID19"[tiab] OR "COVID-19 Pandemic"[tiab] OR "COVID 19 Pandemic"[tiab] OR "COVID-19 Pandemics"[tiab] OR "SARS-CoV-2 Infection"[tiab] OR "SARS-CoV-2 Infections"[tiab] OR "2019-nCoV Disease"[tiab] OR "2019-nCoV Diseases"[tiab] OR "COVID-19 Virus Infection"[tiab] OR "COVID-19 Virus Infections"[tiab] OR "Coronavirus Disease-19"[tiab] OR "COVID-19 Virus Disease"[tiab] OR "2019-nCoV Infection"[tiab] OR "2019-nCoV Infections"[tiab]) |
| Scopus  (n=86) | ( TITLE-ABS-KEY ( "Arthritis, Reactive" OR "Reactive Arthritis" OR "Post-Infectious Arthritis" OR "Post Infectious Arthritis" OR "Reiter Syndrome" OR "Reiter's Disease" OR "Reiter's Syndrome" OR "Reiter Disease" ) AND TITLE-ABS-KEY ( "covid-19" OR "COVID 19" OR "SARS CoV 2 Infection" OR "2019 Novel Coronavirus Disease" OR "2019 Novel Coronavirus Infection" OR "2019 nCoV Disease" OR "COVID 19 Virus Infection" OR "Coronavirus Disease 2019" OR "Coronavirus Disease 19" OR "Severe Acute Respiratory Syndrome Coronavirus 2 Infection" OR "SARS Coronavirus 2 Infection" OR "COVID 19 Virus Disease" OR "2019 nCoV Infection" OR "COVID19" OR "COVID-19 Pandemic" OR "COVID 19 Pandemic" OR "COVID-19 Pandemics" OR "SARS-CoV-2 Infection" OR "SARS-CoV-2 Infections" OR "2019-nCoV Disease" OR "2019-nCoV Diseases" OR "COVID-19 Virus Infection" OR "COVID-19 Virus Infections" OR "Coronavirus Disease-19" OR "COVID-19 Virus Disease" OR "2019-nCoV Infection" OR "2019-nCoV Infections" ) ) |
| Embase  (n=107) | ('reactive arthritis'/exp OR 'reactive arthritis’) AND ('coronavirus disease 2019'/exp OR 'coronavirus disease 2019') |

**S2** The detailed search terms in the databases
