# Supplementary material for: COVID‐associated arthritis after severe and non‐severe COVID‐19: A systematic review
Source: Immun Inflamm Dis. 2023 Oct 11;11(10):e1035. doi: 10.1002/iid3.1035 (PMC10566449; doi:10.1002/iid3.1035)
Supplement: Supplementary file 3 — Supporting information. [file IID3-11-e1035-s003.docx]

**COVID-associated arthritis after severe and non-severe COVID-19: a systematic review**

Mahsa Zarpoosh, Parsa Amirian*

**corresponding author; Email:* [*parsapj@gmail.com*](mailto:parsapj@gmail.com)

*General Practitioner, Kermanshah University of Medical Science (KUMS), Kermanshah, Iran*

**S3(a)** Quality Assessment based on JBI critical appraisal checklist for case series

| Author (ref.) | 1 | 2 | 3 4 5 6 7 | | | | | 8 | 9 10 | | Total score | Total Quality |
| --- | --- | --- | --- | --- | --- | --- | --- | --- | --- | --- | --- | --- |
| Lopez-Gonzalez et al. |  | * | * |  |  |  | * | * | * |  | 5 | satisfactory |
| Vogler et al. | * | * | * | * |  |  | * | * |  |  | 6 | good |
| Visalakshy et al. | * | * | * | * | * | * | * | * | * |  | 9 | excellent |
| Sinha et al. | * | * | * | * | * | * | * | * | * |  | 9 | excellent |
| Pal et al. | * | * | * | * |  | * | * | * | * |  | 8 | excellent |

Scores: Excellent: 8-10 points; Good: 6-7 points; Satisfactory: 4-5 points; Unsatisfactory: 0-3 points

**S3(b)** Quality Assessment based on JBI critical appraisal checklist for case reports

| Author (ref.) | 1 | 2 | 3 4 5 6 7 | | | | | 8 | Total score | Total Quality |
| --- | --- | --- | --- | --- | --- | --- | --- | --- | --- | --- |
| Sinaei et al. | * |  | * |  | * | * |  |  | 4 | satisfactory |
| El Hasbani et al. | * | * | * | * | * | * |  |  | 6 | good |
| Basheikh et al. | * | * | * | * | * | * |  |  | 6 | good |
| Dombret et al. | * | * | * | * | * | * |  |  | 6 | good |
| Shimoyama et al. | * | * | * | * | * | * |  |  | 6 | good |
| Quaytman et al. | * | * | * | * | * | * |  |  | 6 | good |
| Jabalameli et al. |  |  | * | * | * | * |  |  | 4 | satisfactory |
| Ganta et al. | * | * | * | * | * | * |  |  | 6 | good |
| Ruiz-del-Valle et al. | * | * | * | * | * | * |  |  | 6 | good |
| Luceño et al. | * | * | * |  | * | * |  |  | 5 | good |

***Online Resource 4(b)*** *(continued)*

| Author (ref.) | 1 | 2 | 3 4 5 6 7 | | | | | 8 | Total score | Total Quality |
| --- | --- | --- | --- | --- | --- | --- | --- | --- | --- | --- |
| Liew et al. |  |  | * |  | * |  |  |  | 2 | unsatisfactory |
| Talarico et al. |  |  | * |  | * | * |  |  | 3 | satisfactory |
| Danssaert et al. | * | * | * |  | * | * |  |  | 5 | good |
| Parisi et al. |  |  | * | * | * | * |  |  | 4 | satisfactory |
| Sidhu et al. | * | * | * | * | * | * |  |  | 6 | good |
| Fragata et al. |  |  | * |  | * | * |  |  | 3 | satisfactory |
| De Stefano et al. |  | * | * | * | * | * |  |  | 5 | good |
| Houshmand et al. |  |  | * |  | * | * |  |  | 3 | satisfactory |
| Waller et al. |  |  | * |  |  | * |  |  | 2 | unsatisfactory |
| Jali et al. | * | * | * |  | * | * |  |  | 5 | good |
| Mukarram et al. | * | * | * |  | * | * |  |  | 5 | good |
| Salvatierra et al. |  |  | * | * | * | * |  |  | 4 | satisfactory |
| Gibson.M et al. | * | * | * |  | * | * |  |  | 5 | good |
| Saricaoglu et al. | * | * | * |  | * | * |  |  | 5 | good |
| Ono et al. |  |  | * | * | * | * |  |  | 4 | satisfactory |
| Yokogawa et al. | * | * | * | * | * | * |  |  | 6 | good |
| Gasparotto et al. | * | * | * | * | * | * |  |  | 6 | good |
| Alivernini et al. | * | * | * |  | * | * |  |  | 5 | good |
| Sureja et al. |  |  | * | * | * | * |  |  | 4 | satisfactory |
| Shokraee et al. | * | * | * |  | * | * |  |  | 5 | good |
| Ouedraogo et al. | * | * | * | * | * | * |  |  | 6 | good |
| Hønge et al. | * | * | * | * | * | * |  |  | 6 | good |
| Kocyigit et al. | * | * | * | * | * | * |  |  | 6 | good |
| Santacruz et al. |  |  | * | * | * | * |  |  | 4 | satisfactory |
| Di Carlo et al. |  |  | * |  | * | * |  |  | 3 | satisfactory |
| Apaydin et al. | * | * | * | * | * | * |  |  | 6 | good |
| Cincinelli et al. | * | * | * |  | * | * |  |  | 5 | good |
| Dutta et al. |  |  | * | * | * | * |  |  | 4 | satisfactory |
| Colatutto et al. | * | * | * | * | * | * |  |  | 6 | good |
| Coath et al. | * | * | * |  | * | * |  |  | 5 | good |
| Saikali et al. |  |  | * | * | * | * |  |  | 4 | satisfactory |

Scores: Excellent: 7-8 points; Good: 5-6 points; Satisfactory: 3-4 points; Unsatisfactory: 0-2 points

**JBI Critical Appraisal Checklist for Case Series**

1. Were there clear criteria for inclusion in the case series?

2. Was the condition measured in a standard, reliable way for all participants included in the case series?

3. Were valid methods used for the identification of the condition for all participants included in the case series?

4. Did the case series have consecutive inclusion of participants?

5. Did the case series have complete inclusion of participants?

6. Was there clear reporting of the demographics of the participants in the study?

7. Was there clear reporting of clinical information of the participants?

8. Were the outcomes or follow-up results of cases clearly reported?

9. Was there clear reporting of the presenting site(s)/clinic(s) demographic information?

10. Was statistical analysis appropriate?

Moola S, Munn Z, Tufanaru C, Aromataris E, Sears K, Sfetcu R, Currie M, Qureshi R, Mattis P, Lisy K, Mu P-F. Chapter 7: Systematic reviews of etiology and risk. In: Aromataris E, Munn Z (Editors). Joanna Briggs Institute Reviewer's Manual. The Joanna Briggs Institute, 2017. Available from <https://reviewersmanual.joannabriggs.org/>

**JBI Critical Appraisal Checklist for Case Reports**

1. Were the patient’s demographic characteristics clearly described?

2. Was the patient’s history clearly described and presented as a timeline?

3. Was the current clinical condition of the patient on presentation clearly described?

4. Were diagnostic tests or assessment methods and the results clearly described?

5. Was the intervention(s) or treatment procedure(s) clearly described?

6. Was the post-intervention clinical condition clearly described?

7. Were adverse events (harms) or unanticipated events identified and described?

8. Does the case report provide takeaway lessons?

Moola S, Munn Z, Tufanaru C, Aromataris E, Sears K, Sfetcu R, Currie M, Qureshi R, Mattis P, Lisy K, Mu P-F. Chapter 7: Systematic reviews of etiology and risk. In: Aromataris E, Munn Z (Editors). Joanna Briggs Institute Reviewer's Manual. The Joanna Briggs Institute, 2017. Available from <https://reviewersmanual.joannabriggs.org/>
